# The Functional Role of Individual Alpha-Based Frontal Asymmetry in the Processing of Fearful Faces

**DOI:** 10.3389/fpsyg.2020.01412

**Published:** 2020-06-30

**Authors:** Lei Liu, Renlai Zhou

**Affiliations:** ^1^Department of Psychology, College of Teacher Education, Ningbo University, Ningbo, China; ^2^Department of Psychology, School of Social and Behavioral Sciences, Nanjing University, Nanjing, China

**Keywords:** frontal electroencephalography alpha asymmetry, facial expression, fearful face, event-related potential, ERP

## Abstract

The ability to quickly identify fearful faces is important for the activation of defense mechanisms that allow an individual to deal with potential emergencies. This study examined the relationship between frontal electroencephalography (EEG) alpha asymmetry and the processing of congruent and incongruent fearful faces among female participants using event-related potentials (ERPs). Behavioral results showed that individuals with more left frontal EEG alpha asymmetry had shorter response times than individuals with more right frontal EEG alpha asymmetry during the cue-target task. ERP results indicated that, for individuals with more left frontal EEG alpha asymmetry, enhanced N1 reflected more rapid processing of emotional faces in the early stage, and enhanced P3 indicated that these individuals directed more attentional and motivational resources to the evaluation of emotional faces in the late stage. For individuals with more right frontal EEG alpha asymmetry, enhanced N2 indicated that these individuals experienced more conflict for incongruent fearful faces in the late stage. The present findings suggest that frontal EEG alpha asymmetry during resting conditions can reflect individual differences in the processing of congruent and incongruent fearful faces.

## Introduction

Investigation of the factors that underlie individual differences in the evaluation of emotional stimuli continues to be a central focus in the field of affective neuroscience. Fear expression as the fundamental emotional face stimuli plays an important role in human survival and adaptation (Itier et al., 2006; Luo et al., 2010). The ability to quickly identify fearful faces is important in evaluating a dangerous situation and planning an appropriate psychological or behavioral response (Rossignol et al., 2005; Peng et al., 2012). The purpose of the current study was to examine how the processing of fearful faces is related to frontal electroencephalography (EEG) alpha asymmetry during resting conditions, which is a neurophysiological index of emotional processing (Jackson et al., 2003; Kline et al., 2007; Papousek et al., 2012, 2017).

Frontal EEG alpha asymmetry reflects differences in activation of the alpha frequency band (typically 8–13 Hz) of the left and right frontal cortices; there is an inverse relationship between activity within the alpha range and cortical processing (Davidson, 1995; Laufs et al., 2003). Research suggests that frontal EEG alpha asymmetry during resting conditions can moderate the response to emotional stimuli. For example, individuals with more left frontal EEG alpha asymmetry (ILA) have less negative and more positive affect (Tomarken et al., 1992), superior emotional flexibility (Kline et al., 2007; Papousek et al., 2012), more effective emotion regulation (Jackson et al., 2003; Papousek et al., 2017), and lower stress-induced cortisol levels (Quaedflieg et al., 2015), compared to individuals with more right EEG alpha asymmetry (IRA). Recently, Suo et al. (2017) found that ILA had a larger P3 to negative pictures than to positive and neutral pictures, whereas there were no significant ERP differences to negative, positive, and neutral pictures for IRA, suggesting that left-active individuals direct more attentional resources to negative pictures. In addition, Harmon-Jones and Gable (2009) found that greater left frontal-central activation during dessert pictures predicted faster local-target response times after dessert pictures, indicating that greater left frontal-central activation caused narrowing of attention. These findings suggest that frontal EEG alpha asymmetry during resting conditions can reflect individual differences in emotional perception tendencies to emotional stimuli.

Event-related potentials (ERPs) have high temporal resolution and can be used to study the unfolding of emotional processing. A large number of studies have investigated the time course of neural activity underlying the processing of fearful faces using ERPs. Findings indicate that fearful faces elicit a larger P1 (Eimer and Holmes, 2002, 2007; Holmes et al., 2003; Pourtois et al., 2005; Vuilleumier and Pourtois, 2007), a larger P2 (Ashley et al., 2004), and a sustained positive amplitude (Eimer and Holmes, 2002, 2007). Furthermore, some studies have also examined the time course of neural activity underlying the processing of congruent and incongruent fearful faces using ERPs; these studies have examined several ERP components that measure early processing periods and late processing periods, respectively. For example, Peng et al. (2012) showed that for the expression effect (fearful vs. neutral faces), there were differences in early time periods (N1 and P2) between predictable and unpredictable trials, whereas there were no differences in the late time periods (N220–350 and P3). These results reveal that the processing of congruent and incongruent fearful faces differs mainly in the early stage of neural activity after face onset. However, Yang et al. (2012) showed that incongruent fearful faces had larger P2 and N200–300 amplitudes than incongruent neutral faces, whereas there were no differences between congruent fearful and neutral faces for these ERP components. In the early processing period, N1 is associated with early perceptual processing (Peng et al., 2012), and P2 is correlated with increased attention allocation to emotional stimuli (Peng et al., 2012; Yang et al., 2012; Jin et al., 2013). In the late processing period, N2 reflects the monitoring of cognitive interference (Dennis and Chen, 2007; Folstein and Van Petten, 2008; Peng et al., 2012), and P3 is considered to index the attentional and motivational resources allocated to the evaluation of fearful faces (Peng et al., 2012; Ran et al., 2014). The present study explored how frontal EEG alpha asymmetry during resting conditions relates to the processing of congruent and incongruent fearful faces using ERP markers.

In addition, emotional processing is different between women and men. Studies have shown that compared with men, women are more accurate and faster in identifying emotional stimuli (Thayer and Johnsen, 2000; Collignon et al., 2010), have more intense emotional experiences (Lang et al., 1993), and are better able to memorize emotional events (Ros and Latorre, 2010). Furthermore, there are differences in both early and late ERP components between women and men. For the early ERP components, women show larger P1 to subthreshold fearful faces (Lee et al., 2017) and enhanced P2 in response to incongruent negative stimuli (Jin et al., 2013), as compared to men. For the late ERP components, women show larger P3 responses and better memory retrieval for emotional stimuli (Gasbarri et al., 2006), as compared to men.

Given the above, the current study aimed to examine the relationship between frontal EEG alpha asymmetry and the processing of congruent and incongruent fearful faces among female participants using ERP markers. In this study, participants first completed a 2 min resting task and then completed a cue-target task. For the purposes of this study, we were interested in early processing components (e.g., N1 and P2) and late processing components (e.g., N2 and P3) as they relate to the processing of congruent and incongruent fearful faces. Previous studies have indicated that frontal EEG alpha asymmetry during resting conditions can be considered as a neural index of emotional regulation (Jackson et al., 2003; Kline et al., 2007; Papousek et al., 2012, 2017). Relative left lateralization is associated with flexible emotional responses, whereas relative right lateralization is associated with inflexible emotional responses. Thus, we expected ILA to direct more attentional and motivational resources to emotional faces, which would result in enhanced N1 and P3 amplitudes, while IRA were expected to experience more conflict for incongruent emotional faces, resulting in enhanced N2 amplitude. That is, it was expected that frontal EEG alpha asymmetry during resting conditions would reflect individual differences in the processing of congruent and incongruent fearful faces.

## Materials and Methods

### Participants

G ^∗^ Power software was used to calculate the sample size in order to achieve a power of 0.85 at an α level 0.05 with an effect size of 0.30. The output of G ^∗^ Power software indicated that a sample size of 52 was required. As such, 56 healthy female undergraduate students (M = 21.91 years, SD = 2.37 years, age range = 19–28 years) were paid to participate in this study. All participants self-reported that they were right-handed with normal or corrected-to-normal vision and had no neurological or psychological disorders. All participants completed the Beck Depression Inventory (BDI) and Beck Anxiety Inventory (BAI), and all participants were suitable for the experiment because their scores on the BDI and BAI were within the normal range. All participants gave their written informed consent, and the study was approved by the local ethics committee of Ningbo University.

### Experimental Materials

#### Emotional Questionnaire Materials

The BDI is a 21-item self-report measure designed to assess depression (Beck et al., 1979). The Chinese BDI scale has a split-half reliability coefficient of 0.88 and a Cronbach’s alpha coefficient of 0.89 (Zhang et al., 1990). The BAI is a 21-item self-report measure designed to assess anxiety (Beck et al., 1988). The Chinese BAI has good reliability, with a Cronbach’s alpha coefficient of 0.95 (Zheng et al., 2002).

#### Emotional Face Materials

In total, 60 emotional faces were selected from the Chinese Facial Affective Picture System Database (Wang and Luo, 2005); these included 15 fearful male and 15 fearful female faces (valence: M = 2.62, SD = 0.32; arousal: M = 6.71, SD = 0.99) as well as 15 neutral male and 15 neutral female faces (valence: M = 4.68, SD = 0.32; arousal: M = 4.22, SD = 0.28). There were significant differences between fear faces and neutral faces in terms of valence [*F*(1,58) = 596.49, *p* < 0.001] and arousal [*F*(1,58) = 172.06, *p* < 0.001] (please see Peng et al., 2012).

### Procedure

**FIGURE 1:**
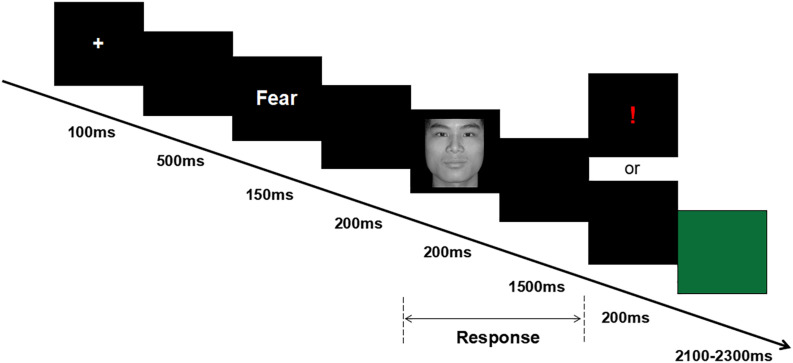
Overview of a representative experimental trial.

After attending the lab, participants signed the informed consent form and completed the emotional questionnaires. Then, participants were seated in an acoustically and electrically shielded examination chamber, approximately 100 cm from a computer screen, and electrodes were attached. (1) Participants were asked to complete a 2 min resting task, in which recording of resting EEG was obtained; the 2 min resting task included 1 min eyes open (O) and 1 min eyes closed (C). Two sequences were used, O–C–C–O and C–O–O–C; the presentation of these sequences was balanced between the subjects. (2) Participants were asked to complete the cue-target task, which was a modified version of the task used by Peng et al. (2012) (see Figure 1). During the cue-target task, each trial started with a white cross for 100 ms. After a 500-ms black blank, the cue word (i.e., the word “fear” or “neutral”) was presented for 150 ms. After another 200-ms black blank, the target face was presented for 200 ms, followed by a black blank whose longest duration was 1,500 ms. Half of the participants were instructed to use their left hand to press the “F” key if a fearful face was shown or to use their right hand to press the “J” key if a neutral face was shown, whereas the other half of the participants were instructed to use a reversed key arrangement. For incorrect or invalid responses, an exclamation mark was displayed for 200 ms; otherwise, the black blank remained for another 200 ms. Finally, a green blank was presented for a random duration of 2,100–2,300 ms, allowing the participant to relax for a while. In each trial, the chance of a consistent prime-face sequence was 50%; there were four conditions in the experiment: “fear” word-fear face, “fear” word-neutral face, “neutral” word-fear face, and “neutral” word-neutral face. The experiment consisted of 180 formal trials, divided over two blocks.

E-prime 2.0 software was used to present the 2 min resting task and the cue-target task. All stimuli were presented in the center of a 17-inch LCD screen (resolution 1024 × 768, refresh rate 60 Hz).

### EEG Recording and Analysis

Electroencephalography data were recorded using a NeuroScan recorder with a NuAmps amplifier. The electrode cap contained 40 Ag/AgCl electrodes, which were positioned according to the International 10–20 system. EEG signals were acquired by a DC model with a sampling rate of 1,000 Hz and a bandwidth of 100 Hz. Vertical and horizontal electrooculograms (EOGs) were recorded; the left mastoid electrode served as the reference. The impedance of all electrodes was kept under 5 kΩ.

#### EEG Data Analysis

Electroencephalography asymmetry measures were taken from the 2-min resting task. Neuroscan 4.3 software was used to analyze the EEG data. According to previous studies (Feng et al., 2012; Papousek et al., 2012; Huang et al., 2015; Suo et al., 2017; Zhou and Liu, 2017), all data were inspected visually to eliminate intervals in which ocular or muscle artifacts occurred. Only participants who had at least 30 s of artifact-free data in the recording periods were included in the final sample (*n* = 56). Offline analysis of EEG signals was re-referenced to the Cz electrode^1^ and was filtered using a 30 Hz bandwidth (24 dB/octave slope). Power spectra were derived by fast Fourier transform with a Hamming window (epoch length 1 s, 50% overlap) for the 2-min resting task. For consistency with previous research (Papousek et al., 2012; Suo et al., 2017), we focused on the alpha band (8–13 Hz) in the frontal electrodes F3 and F4. A laterality coefficient (LC) indexing relative left- versus right-sided activation was used. EEG LC values were computed as follows: LC = [(*L* − *R*)/(*L* + *R*)] × 100. Positive values indicate higher alpha activity in the left compared to the right hemisphere.

#### ERP Data Analysis

The offline analysis of EEG signals was re-referenced to the mean of the left and right mastoids and was filtered using a 0.05- to 30-Hz bandwidth (24 dB/octave slope). Vertical and horizontal EOGs were filtered out according to the computation rule commonly used in ERP studies (Gratton et al., 1983). The artifact rejection criterion was an amplitude of ±100 μV. Table 1 shows the average trial number of four conditions for ILA and IRA. The EEG was averaged by channel and time window from 100 ms before prime cue to 1,400 ms after prime cue. The 100-ms interval before prime cue onset served as the baseline interval. According to previous studies (Folstein and Van Petten, 2008; Luo et al., 2010; Peng et al., 2012; Yang et al., 2012), for the grand-mean ERP waveforms, we measured the mean amplitudes of N1 (480–520 ms after prime onset or 130–170 ms after face onset) and P2 (540–600 ms after prime onset or 190–250 ms after face onset) over the anterior (Fz, FCz, and Cz) regions, and the mean amplitudes of N2 (630–730 ms after prime onset or 280–380 ms after face onset) and P3 (830–1,130 ms after prime onset or 480–780 ms after face onset) over the anterior (Fz, FCz, and Cz) and posterior (Pz and CPz) regions.

**TABLE 1:** The average trial number of the four conditions for ILA and IRA, respectively.

	ILA(*n* = 28) *M* (Minimum, Maximum)	IRA(*n* = 28) *M* (Minimum, Maximum)
FC-FF	42 (27, 45)	40 (24, 45)
FC-NF	41 (31, 45)	40 (24, 45)
NC-FF	42 (30, 45)	39 (20, 45)
NC-NF	41 (27, 45)	39 (22, 45)

*FC: fear cue; NC: neutral cue; FF: fearful face; NF: neutral face.*

### Statistics

According to a median split of frontal EEG alpha asymmetry scores during the 2-min resting task (Papousek et al., 2012; Suo et al., 2017), individuals were divided into two groups: ILA and IRA. The behavioral measures (the accuracy rates and the response times) were analyzed using a 2 (prime cue: “neutral” word vs. “fear” word) × 2 (expression type: neutral face vs. fear face) × 2 (group: ILA vs. IRA) mixed factor ANOVA, in which prime cue and expression type were the within-subject factors and group was the between-subjects factor. Then, for N1 and P2, a 2 (prime cue: “neutral” word vs. “fear” word) × 2 (expression type: neutral face vs. fear face) × 2 (group: ILA vs. IRA) fixed-measures ANOVA was performed, in which prime cue and expression type were the within-subjects factors and group was the between-subjects factor. For N2 and P3, a 2 (prime cue: “neutral” word vs. “fear” word) × 2 (expression type: neutral face vs. fear face) × 2 (electrode: anterior vs. posterior) × 2 (group: ILA vs. IRA) fixed-measures ANOVA was performed, in which prime cue, expression type, and electrode were the within-subjects factors and group was the between-subjects factor. The significance levels were set at 0.05.

## Results

In this section, we first report the behavioral results. Then, the ERP results are reported. For the sake of brevity, the statistical effects that did not reach significance are omitted.

### Behavioral Results

Before statistical analysis, the no-response trials were removed. Then, subjects were divided into two groups (IRA and ILA) based on a median split of baseline asymmetry. Table 2 shows the mean ages, mean scores on the emotional questionnaires (BAI and BDI), and the LCs for the ILA and IRA groups.

**TABLE 2:** The mean age, scores on the emotional questionnaires (BAI and BDI), and laterality coefficient (LC) for ILA and IRA, respectively.

	ILA(*n* = 28) *M*(SD)	IRA(*n* = 28) *M*(SD)	*t(p)*
Age	22.07 (2.07)	21.75 (2.66)	0.50 (0.616)
BAI	26.21 (5.32)	26.64 (6.17)	0.28 (0.782)
BDI	6.57 (6.31)	7.75 (7.03)	−0.66(0.512)
LC	−16.07(11.23)	11.14 (14.46)	−7.86(0.000)

**FIGURE 2:**
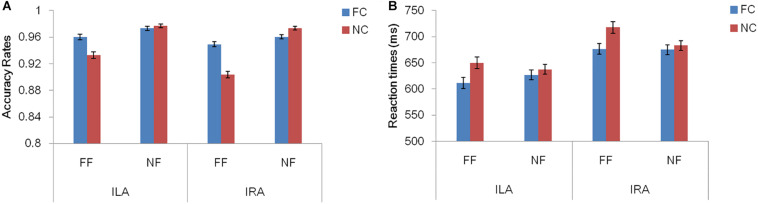
The accuracy rates **(A)** and response times **(B)** for the four conditions, for ILA and IRA, respectively. FC: fear cue; NC: neutral cue; FF: fearful face; NF: neutral face.

Table 3 and Figure 2 show the accuracy rates and the response times in the four conditions, for ILA and IRA, respectively. Table 4 shows the statistical results for the behavioral data, for the ILA and IRA groups.

**TABLE 3:** The means and standard deviations of the behavioral data (accuracy rate and response time) for ILA and IRA, respectively.

	ILA(*n* = 28) *M*(SD)	IRA(*n* = 28) *M*(SD)
**ACC**		
FC-FF	0.96 ± 0.04	0.95 ± 0.04
FC-NF	0.97 ± 0.03	0.96 ± 0.04
NC-FF	0.93 ± 0.04	0.90 ± 0.06
NC-NF	0.98 ± 0.02	0.97 ± 0.03
**RT**		
FC-FF	611.49 ± 87.25	676.63 ± 125.94
FC-NF	626.84 ± 84.90	675.16 ± 109.10
NC-FF	649.95 ± 93.17	717.69 ± 132.91
NC-NF	637.52 ± 81.76	683.33 ± 114.21

**TABLE 4:** The statistical results for the behavioral data (accuracy rate and response time) for ILA and IRA, respectively.

	*F*	*p*	η^2^
**Accuracy rate**			
PC	16.81	0.000	0.237
ET	55.19	0.000	0.505
G	2.96	0.091	0.052
PC × ET	33.94	0.000	0.386
G × PC	0.42	0.522	0.008
G × ET	1.67	0.202	0.030
G × PC × ET	3.194	0.080	0.056
**Response time**			
PC	54.50	0.000	0.502
ET	2.30	0.135	0.041
G	4.39	0.041	0.075
PC × ET	13.67	0.001	0.202
G × PC	0.00	0.995	0.000
G × ET	3.19	0.080	0.056
G × PC × ET	0.10	0.757	0.002

*PC: Prime Cue; ET: Expression Type; G: Group.*

For the accuracy rates, the main effect of the prime cue was significant, *F*(1,54) = 16.81, *p* < 0.001, η^2^ = 0.237, indicating that the accuracy rate for the word “fear” was higher than for the word “neutral.” The main effect of expression type was significant, *F*(1,54) = 55.19, *p* < 0.001, η^2^ = 0.505, indicating that the accuracy rate for fear faces was lower than for neutral faces. The interaction effect of prime cue × expression type was significant, *F*(1,54) = 33.94, *p* < 0.001, η^2^ = 0.386. The simple effect analysis of prime cue × expression type showed that the accuracy rate for fear faces when the prime cue was the word “fear” was higher than that when the prime cue was the word “neutral” (*p* < 0.001), whereas there was no significant difference between neutral faces when the prime cue was the word “fear” and when the prime cue was the word “neutral” (*p* = 0.068).

For response times, the main effect of the prime cue was significant, *F*(1,54) = 54.50, *p* < 0.001, η^2^ = 0.502, indicating that reaction times to the word “fear” were shorter than to the word “neutral.” The main effect of group was significant, *F*(1,54) = 4.39, *p* = 0.041, η^2^ = 0.075, indicating that the reaction times of the ILA group were shorter than those of the IRA group. The interaction effect of prime cue × expression type was significant, *F*(1,54) = 13.67, *p* = 0.001, η^2^ = 0.202. The simple effect analysis of prime cue × expression type showed that when the prime cue was the word “neutral,” the reaction times for fear faces were longer than neutral faces (*p* = 0.003), whereas there was no significant difference between fear faces and neutral faces when the prime cue was the word “fear” (*p* = 0.256).

### ERP Results

**FIGURE 3:**
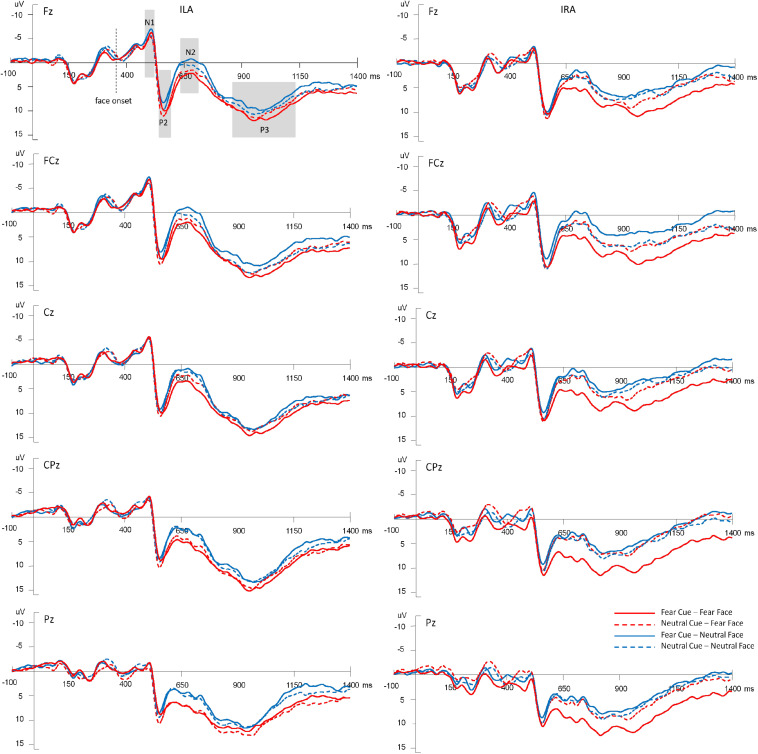
The average amplitudes of the four conditions at Fz, FCz, Cz, CPz, Pz electrodes for ILA and IRA, respectively.

Figure 3 shows the average amplitudes in the four conditions at Fz, FCz, Cz, CPz, Pz electrodes for ILA and IRA, respectively. Table 5 shows the means and standard deviations of ERP data for ILA and IRA, and Table 6 shows the statistical results for the ERP data for ILA and IRA.

**TABLE 5:** The means and standard deviations of the ERP data (N1, P2, N2, and P3) for ILA and IRA, respectively.

	ILA(*n* = 28) *M*(SD)	IRA(*n* = 28) *M*(SD)
**N1**		
FC-FF	−5.00 ± 4.38	−1.47 ± 5.05
FC-NF	−5.23 ± 6.22	−2.89 ± 4.97
NC-FF	−4.19 ± 3.91	−2.82 ± 5.25
NC-NF	−4.36 ± 3.94	−1.74 ± 4.77
**P2**		
FC-FF	9.30 ± 5.46	9.63 ± 6.99
FC-NF	7.67 ± 5.96	8.02 ± 6.49
NC-FF	9.94 ± 5.29	9.54 ± 8.75
NC-NF	8.63 ± 4.79	9.38 ± 6.95
** Anterior N2**		
FC-FF	3.51 ± 5.83	4.84 ± 7.08
FC-NF	0.58 ± 6.05	1.35 ± 6.23
NC-FF	2.80 ± 5.57	2.55 ± 9.12
NC-NF	1.68 ± 4.83	2.45 ± 6.87
**Posterior N2**		
FC-FF	7.01 ± 4.69	8.47 ± 5.36
FC-NF	4.21 ± 5.19	4.34 ± 5.37
NC-FF	6.92 ± 5.03	5.03 ± 7.40
NC-NF	4.71 ± 4.33	4.83 ± 6.01
**Anterior P3**		
FC-FF	11.93 ± 6.06	8.67 ± 12.05
FC-NF	10.06 ± 5.66	4.55 ± 12.57
NC-FF	11.50 ± 5.85	6.30 ± 12.22
NC-NF	11.09 ± 6.19	5.78 ± 11.82
**Posterior P3**		
FC-FF	12.04 ± 4.95	9.97 ± 10.37
FC-NF	10.39 ± 5.43	5.70 ± 10.25
NC-FF	12.36 ± 5.71	6.47 ± 10.21
NC-NF	10.85 ± 6.06	6.37 ± 9.74

**TABLE 6:** The statistical results for the ERP data (N1, P2, N2, and P3) for ILA and IRA, respectively.

	*F*	*p*	η^2^
**N1**			
PC	0.71	0.404	0.013
ET	0.26	0.609	0.005
G	4.85	0.032	0.082
PC × ET	3.77	0.057	0.065
G × PC	1.13	0.293	0.020
G × ET	0.01	0.960	0.000
G × PC × ET	3.41	0.070	0.059
**P2**			
PC	2.92	0.093	0.051
ET	8.20	0.006	0.132
G	0.03	0.870	0.001
PC × ET	1.41	0.240	0.025
G × PC	0.04	0.848	0.001
G × ET	0.51	0.477	0.009
G × PC × ET	0.58	0.449	0.011
**N2**			
PC	0.87	0.355	0.016
ET	25.55	0.000	0.321
E	67.88	0.000	0.557
G	0.05	0.825	0.001
PC × ET	10.36	0.002	0.161
PC × E	5.37	0.024	0.090
G × PC	1.92	0.171	0.034
G × ET	0.11	0.739	0.002
G × E	0.80	0.376	0.015
ET × E	2.63	0.111	0.046
PC × ET × E	0.82	0.369	0.015
G × PC × ET	2.65	0.110	0.047
G × PC × E	5.61	0.021	0.094
G × ET × E	0.04	0.846	0.001
G × PC × ET × E	5.41	0.024	0.091
**P3**			
PC	0.39	0.535	0.007
ET	12.69	0.001	0.190
E	0.67	0.418	0.012
G	4.70	0.035	0.080
PC × ET	5.61	0.021	0.094
PC × E	3.32	0.074	0.058
G × PC	1.66	0.203	0.030
G × ET	0.78	0.381	0.014
G × E	0.17	0.684	0.003
ET × E	0.34	0.561	0.006
PC × ET × E	0.64	0.429	0.012
G × PC × ET	2.44	0.124	0.043
G × PC × E	5.00	0.030	0.085
G × ET × E	1.14	0.291	0.021
G × PC × ET × E	4.11	0.048	0.071

*PC: Prime Cue; ET: Expression Type; G: Group; E: Electrode.*

For N1, the main effect of group was significant, *F*(1,54) = 4.85, *p* = 0.032, η^2^ = 0.082, indicating that ILA had larger N1 amplitudes than IRA.

For P2, the main effect of expression type was significant, *F*(1,54) = 11.29, *p* = 0.001, η^2^ = 0.173, indicating that fear faces induced larger P2 amplitudes than neutral faces.

For N2, the main effect of expression type was significant, *F*(1,54) = 25.55, *p* < 0.001, η^2^ = 0.321, indicating that fear faces induced smaller N2 amplitudes than neutral faces. The main effect of electrode was significant, *F*(1,54) = 67.88, *p* < 0.001, η^2^ = 0.557, indicating that the anterior region induced greater N2 amplitudes than the posterior region. The interaction effect of prime cue × expression type was significant, *F*(1,54) = 10.36, *p* = 0.002, η^2^ = 0.161. The interaction effect of prime cue × electrode was significant, *F*(1,54) = 5.37, *p* = 0.024, η^2^ = 0.090. The interaction effect of prime cue × electrode × group was significant, *F*(1,54) = 5.61, *p* = 0.021, η^2^ = 0.094. The interaction effect of prime cue × expression type × group × electrode was significant, *F*(1,54) = 5.41, *p* = 0.024, η^2^ = 0.091. The simple effect analysis of prime cue × expression type × electrode × group showed that for IRA, fear faces when the prime cue was the word “neutral” induced greater N2 amplitudes than when the prime cue was the word “fear” (*p*’s < 0.05). However, there were no significant differences for ILA (*p*’s > 0.05).

For P3, the main effect of expression type was significant, *F*(1,54) = 12.69, *p* = 0.001, η^2^ = 0.190, indicating that fear faces induced greater P3 amplitudes than neutral faces. The main effect of group was significant, *F*(1,54) = 4.68, *p* = 0.035, η^2^ = 0.080, indicating that ILA had greater P3 amplitudes than IRA. The interaction effect of prime cue × expression type was significant, *F*(1,54) = 5.61, *p* = 0.021, η^2^ = 0.094. The interaction effect of prime cue × electrode × group was significant, *F*(1,54) = 4.99, *p* = 0.030, η^2^ = 0.085. The interaction effect of prime cue × expression type × electrode × group was significant, *F*(1,54) = 4.11, *p* = 0.048, η^2^ = 0.071. The simple effect analysis of prime cue × expression type × electrode × group showed that fear faces induced greater P3 amplitudes when the prime cue was the word “fear” than when the prime cue was the word “neutral” for the IRA group only (*p*’s < 0.05). However, there were no significant differences in the ILA group (*p*’s > 0.05). Another simple effect analysis method showed that the ILA group had a larger P3 than the IRA group for the word “neutral,” regardless of whether it was followed by fear faces or neutral faces, and for the word “fear” followed by neutral faces (*p*’s < 0.05). However, there was no significant difference between ILA and IRA for the word “fear” followed by fear faces (*p* > 0.05).

## Discussion

The present study examined whether frontal EEG alpha asymmetry during resting conditions is related to the processing of congruent and incongruent fearful faces among female participants. Behaviorally, we found that the IRA group had longer reaction times than the ILA group during the cue-target task. The ERP results showed that there was a modulating effect of frontal EEG alpha asymmetry on congruent and incongruent fearful faces in N1, N2, and P3 time intervals.

The behavioral results showed that the accuracy of fearful faces when the prime cue word was “fear” was higher than when the prime cue word was “neutral.” These results indicated that fearful faces processing was influenced by anticipation that congruent prime cue had higher accuracy than incongruent prime cue for fearful faces. Some studies have shown “negative bias” for the processing of emotional information, in which negative stimuli are often quicker to attract attention and priority in mental processing (Smith et al., 2006; Yang et al., 2010). In the present study, the word “fear” was considered as a negative stimulus that would attract more attentional resources, thus helping participants to better judge subsequent fearful faces. Furthermore, the reaction time results showed that reactions times for fearful faces were longer than for neutral faces when the prime cue word was “neutral.” When the prime cue word was “neutral,” incongruent fearful faces produced more cognitive conflict than congruent neutral faces, leading to longer response times. Further, the behavioral results showed that the average reaction time of the IRA group was longer than that of the ILA group. Frontal EEG alpha asymmetry can be considered as an index of emotional regulation. Research has shown that IRA has less effective emotion regulation compared with ILA (Jackson et al., 2003; Papousek et al., 2017). Therefore, IRA must devote more time to evaluate threat cues or stimuli, resulting in longer reactions times in the cue-target task.

The ERP results indicated that ILA had larger N1 amplitudes than IRA. Previous studies have shown that N1 is associated with early perceptual processing (Pourtois et al., 2000; Peng et al., 2012). N1 serves as a rapid detector and predictor of potential information based on coarse aspects of input; this detection is valuable for recognizing and analyzing threatening information (Bar et al., 2006). The results of the present study suggest that ILA can detect emotional face stimuli faster than IRA in the early stage.

Further, fearful faces, when the prime cue word was “neutral,” induced greater N2 amplitudes than when the prime cue word was “fear,” among the IRA group only. Previous studies have shown that the N2 component is related to conflict monitoring. For example, N2 is sensitive to the degree of conflict between response alternatives in the flanker task (Kopp et al., 1996; Van Veen and Carter, 2002; Folstein and Van Petten, 2008). Therefore, we suggest that N2 may reflect the monitoring of cognitive interference. In the present study, there was more conflict for incongruent fearful faces than for congruent fearful faces, leading to larger N2 amplitudes for the IRA group. However, ILA exhibits superior emotional flexibility (Kline et al., 2007; Papousek et al., 2012) and more effective emotion regulation (Jackson et al., 2003; Papousek et al., 2017). For example, Jackson et al. (2003) found that ILA displayed attenuated startle magnitude after the offset of negative stimuli, reflecting an automatic emotion regulation process aimed at reducing negative affectivity. Recently, research showed that ILA prefers reappraisal over suppression to regulate negative events (Papousek et al., 2017). Thus, there was no significant difference in N2 amplitude between congruent and incongruent fearful faces for the ILA group.

In addition, the ILA group exhibited larger P3 amplitudes than the IRA group for the word “neutral,” regardless of whether it was followed by fear faces or neutral faces, and for the word “fear” when followed by neutral faces. In studies using emotional stimuli, P3 has been summarized as reflecting the allocation of limited resources toward motivationally salient environment stimuli, in which motivationally relevant stimuli (e.g., emotional stimuli) naturally and automatically arouse and direct attentional and motivational resources (Hajcak et al., 2010; Eddy et al., 2015). According to this, ILA can automatically direct attention and motivation to emotional face stimuli, as compared with IRA. Furthermore, for ILA, there was no significant difference in P3 amplitude between fearful faces followed by the word “neutral” and those followed by the word “fear”; there was also no significant difference in P3 amplitude between neutral faces followed by the word “neutral” and those followed by the word “fear.” Considering that emotional P300 effects reflect rapid attention to emotional stimuli, and are associated with improved processing efficiency (Öhman et al., 2001; Hajcak et al., 2010; Eddy et al., 2015), these results indicate that ILA directed more attentional and motivational resources to the evaluation of congruent and incongruent emotional face stimuli. However, for IRA, congruent fearful faces induced greater P3 amplitudes than incongruent fearful faces, whereas there was no significant difference between congruent and incongruent neutral faces. These results indicate that, for IRA, attentional and motivational resources were directed to the evaluation of fearful faces only when the prime cue word was “fear.”

The present study suggests that frontal EEG alpha asymmetry during resting conditions is associated with the processing of congruent and incongruent fearful faces. The neuro-laterality models of affect and psychopathology assume that the left and right frontal cortical hemispheres are differentially involved in processes modulating affective responses to emotional challenges (Davidson, 1998; Eippert et al., 2007; Harmon-Jones et al., 2010). It has been proposed that greater left frontal EEG activity during resting conditions is associated with greater affective flexibility as compared to asymmetry in favor of the right hemisphere (Papousek et al., 2012). This is consistent with the present findings indicating that relative activation intensity of the left frontal cortex and right frontal cortex during resting conditions is sensitive to the processing of congruent and incongruent fearful faces. To our knowledge, this study is the first to demonstrate such a link between frontal EEG alpha asymmetry during resting conditions and the processing of fearful faces.

A potential limitation of the present study is that it is unclear how frontal EEG alpha asymmetry during resting conditions relates to the processing of congruent and incongruent fearful faces. A previous study found that fear emotion induced by fear stimuli increased activation of the frontal cortex. With the increased frontal cortical activity, there was a downward trend in amygdala activation (Goldstein et al., 2010). Future research should assess whether the frontal cortex affects activation of the amygdala, thereby modulating the processing of congruent and incongruent fearful faces. The second limitation is that this study aimed to examine whether frontal EEG alpha asymmetry during resting conditions is associated with the processing of congruent and incongruent fearful faces among female participants only. One gender was chosen given that emotional processing is reportedly different between women and men (Thayer and Johnsen, 2000; Collignon et al., 2010; Jin et al., 2013; Lee et al., 2017). Future research needs to investigate whether there is a gender difference in their connections. Finally, there were several methodological limitations of this study. First, based on previous studies (Papousek et al., 2012; Suo et al., 2017), the present study created two artificial groups based on a median split of frontal alpha asymmetry; this may decrease the statistical and explanatory power of the study. Second, the number of incorrect responses was not very high in the current study, and the correct and incorrect trials were pooled for ERP analyses. This approach might not be optimal for assessment of amplitude variation in response to congruent and incongruent stimuli for the N2 component, which is usually investigated only for correct trials.

The present study suggests a relationship between frontal EEG alpha asymmetry and the processing of congruent and incongruent fearful faces. In this study, ILA quickly processed the emotional faces in the early stage and directed more attentional and motivational resources to the evaluation of the emotional faces in the late stage, while IRA experienced more conflict for incongruent fearful faces in the late stage and longer reaction times during the cue-target task. Therefore, frontal EEG alpha asymmetry during resting conditions can reflect individual differences in the processing of congruent and incongruent fearful faces.

## Data Availability Statement

The datasets generated for this study are available on request to the corresponding author.

## Ethics Statement

The studies involving human participants were reviewed and approved by the Ethic Committee at the Department of Psychology, Ningbo University. The patients/participants provided their written informed consent to participate in this study. Written informed consent was obtained from the individual(s) for the publication of any potentially identifiable images or data included in this article.

## Author Contributions

Both authors developed the ideas and wrote the manuscript.

## Conflict of Interest

The authors declare that the research was conducted in the absence of any commercial or financial relationships that could be construed as a potential conflict of interest.

## Footnotes

1 Cz reference has been utilized more often in the EEG asymmetry literature than other reference montages (see Coan and Allen, 2003; Coan and Allen, 2004; for review). Therefore, the present study used the Cz reference to analyze the EEG data. In addition, there was a significant correlation between frontal alpha asymmetry scores when using the Cz reference and frontal alpha asymmetry scores when using the mean reference of the left and right mastoids (*r* = 0.695, *p* < 0.001). This result suggests that frontal alpha asymmetry results did not change when using linked mastoids as a reference compared to when using Cz as a reference.
